# Book Review: The Genius Within: Smart Pills, Brain Hacks and Adventures in Intelligence

**DOI:** 10.3389/fpsyg.2018.01930

**Published:** 2018-10-09

**Authors:** Matthew J. Buchan

**Affiliations:** Department of Pharmacology, University of Oxford, Oxford, United Kingdom

**Keywords:** cognition, intelligence, cognitive enhancers, ethics, legislation

The off-label use of nootropics by the healthy has always been surrounded by controversy. However, recent surges in their use across society (Maier et al., 2018), and the emerging popularity of DIY electrical stimulation techniques (Schuijer et al., 2017), have brought the questions surrounding cosmetic neuroscience sharply into focus. David Adam (2018), in his book, *The Genius Within: Smart pills, brain hacks, and adventures in intelligence*, seeks to address the efficacy of cognitive enhancement, and ask questions of society and legislators as to the extent to which we are ready to adopt their use.

The pursuit of consensus within the ethical battleground of cognitive enhancement typically sticks at a point of definition. The recurring moral potholes of efficacy, safety, accessibility, and fairness are generally debated *ad nauseum*, often with disregard for the tension between bio-liberal arguments (i.e., that the use of cognitive enhancers is not imposed upon anyone by society) and the reality of their use in competitive situations, before the consistent conclusion—that further research is needed to better define the title concepts.

Cognition can be broadly defined as mental performance, which itself is not monolithic but constitutes a number of distinctive abilities relating to the acquisition of knowledge and understanding. Intelligence, in turn, can be taken to mean the acquisition and application of such abilities. This of course leaves sufficient room for interpretation dependent upon the socio-cultural context and the weight you ascribe to its genetic component (or lack thereof) (Sternberg and Griggorenko, 2004; Hill et al., 2018). However, common amongst cultures and belief systems with respect to intelligence has been the remarkably dogged determination to reduce human beings to immutable scores and labels, such as IQ.

With a laudable degree of acerbity, the author sidesteps this minefield of posturing at the outset, accepting that intelligence is at least partly dictated by genes, and that IQ scores, despite their reductionist tendencies, do seem to be a “reliable proxy for what most of us would consider to be intelligence.” What follows is a thoroughly entertaining journey of discovery, as the author puts a regime of cognitive enhancement to the test, attempting to cheat his way into the priggish smart club, Mensa (which incidentally is Mexican slang for “stupid woman”).

The author regularly strays into a number of illustrative anecdotes which serve to give an entertaining grounding in the history of intelligence as a concept. Of particular note is the story of Walt Whitman, who donated his brain to the American Anthropometric Society (a so-called “brain club” dedicated to studying the brains of clever people) who, upon his death, subsequently dropped it.

Before he could embark on his experiment of self-enhancement, the author sat the Mensa exam in order to determine his baseline IQ. Rather anti-climactically, he passed the first time, leaving him with no other option than to aim to improve his score. Leaving a year before the second attempt in order to counteract any effects of repetition bias proved to be a convenient amount of time to experiment with a number of enhancement technologies. Time which, it became clear, is necessary if one were to need to, for example: find a lab willing to verify the contents of your black market drugs order, track down someone willing to let you use their DIY brain stimulation headset made from an American Football helmet and some batteries, or gather enough data points to make a robust assessment of the influence of Modafinil upon unforced errors in a weekly squash fixture. Fifteen months later, bolstered by some pills and a $55 electric stimulator, the author was indeed able to boost his Mensa scores. Helpfully concluding that his experiment was “not scientific” and “generated no reliable data,” he turned his gaze back toward the ethical arena.

Societal attitudes toward cognitive enhancement largely reflect perceptions of safety, alongside some form of subjective utility. A proponent of enhancement could make the case that it is a lifestyle choice, akin to smoking, whereby someone chooses to do something with known negative consequences. However, without an understanding of the mechanisms and potential side-effects of a given enhancer, it is difficult to set an ethical baseline, nor indeed establish their true efficacy. This fact is certainly not helped by the popularization of simplistic conceptualizations (for example, linking single neurotransmitters to single cognitive functions) which misrepresent the complexity of the neuronal and behavioral mechanisms involved (Husain and Mehta, 2011). With respect to currently licensed nootropics, given that they are effective for a number of people (with side effects that are sufficiently negligible as to legitimize their prescription) at least some of them are sure to be effective for at least some healthy people. Increasing public acceptance will no doubt bolster the resolve of pharmaceutical companies that have, until now, been far too squeamish to broach cosmetic neuroscience.

Therefore, a careful balance must be struck in order to avoid one of two scenarios. Firstly, an unfounded superstition of cognitive enhancers engendered by premature legislation acting to prohibit their use, inevitably resulting in an entrenched black market and the continued failure to control their use; or secondly, a new flavor of social stratification, characterized by one's access to cognitive enhancers, as the result of a laissez-faire approach to regulation. Sadly, the quixotic view of a super-smart, super-egalitarian society founded upon the universal distribution of smart pills doesn't seem the most likely outcome. With this in mind, scientists and physicians must drive legislators—both to dispel myths, but also to encourage proper discourse.

As the author puts it: “it may be too early to provide answers… [with respect to the use of cognitive enhancers] but it is not too early to ask the questions.” “The Genius Within” is a rigorous and entertaining jaunt through cosmetic neuroscience, brimming with sarcasm, which effectively highlights the mounting requirement for research funding and considered legislation with respect to the effects of cognitive enhancement upon society.

## Author contributions

The author confirms being the sole contributor of this work and approved it for publication.

### Conflict of interest statement

The author declares that the research was conducted in the absence of any commercial or financial relationships that could be construed as a potential conflict of interest.
